# Comparison Analysis of Roundness Measurement of Small Cylindrical Workpieces with Different Styluses

**DOI:** 10.3390/s24123819

**Published:** 2024-06-13

**Authors:** Borong Wu, Chuang Zeng, Qiaolin Li

**Affiliations:** 1 Chongqing University-University of Cincinnati Joint Co-op Institute, Chongqing University, Chongqing 400044, China; 20206107@cqu.edu.cn; 2 Shenzhen International Graduate School, Tsinghua University, Shenzhen 518055, China; zengc21@mails.tsinghua.edu.cn

**Keywords:** roundness measurement, small cylindrical workpieces, ruby ball stylus, diamond stylus, stitching linear scan method

## Abstract

To investigate the high-accuracy roundness metrology of a needle roller 1.5 mm in diameter and 5.8 mm in length using the stitching linear scan method, a ruby ball stylus with a tip radius of 150 μm and a diamond stylus with a tip radius of 2 μm were employed to perform experiments under the same conditions. The precision coordinate data, derived from the needle roller’s cross-sectional circumference, were segmented into uniform eighths, each scanned with the stylus of a roughness measuring machine. The roundness profile of the needle roller was obtained by stitching the arc profiles, which were characterized according to the precision coordinate data of the arcs. The cross-correlation function, Euclidean distance, residual sum of squares, position error, and curvature of the measured arcs were used to evaluate the results, which can reflect the performance of the stylus. A comparison of the results obtained using the ruby ball stylus versus the diamond stylus demonstrates the ruby ball stylus’ greater suitability for use in the roundness metrology of the needle roller bearing examined in this paper.

## 1. Introduction

As fundamental components in manufacturing processes, cylindrical workpieces are pivotal in various industrial fields, including agricultural machinery, intelligent automobiles, additive manufacturing, metallurgical industries, marine vessels, and microelectronic processing [1,2,3,4,5,6]. Their multifunctionality makes them indispensable components within mechanical systems, such as gears, rotating cylinders, crankshafts, and other precision machines [7,8,9]. Among these, roundness serves as a critical quality control parameter that significantly influences the functionality, performance, and lifetime of these components [10,11,12]. An accurate roundness measurement not only validates compliance with design specifications but also has a direct impact on product performance [13]. However, current measurement techniques have limitations, particularly when it comes to accurately evaluating small workpieces. Common roundness measurement methods include the two-point method and the use of roundness gauges. These methods rely on the measurement personnel’s skill and are susceptible to errors caused by workpiece eccentricity and tilt, especially as the dimensions of the cylindrical workpiece being measured decrease, complicating the accuracy of the roundness measurement. Workpieces less than 3 mm in diameter present particular difficulties in this regard [14,15,16].

Over the years, many roundness measurement methods have been proposed. For example, Ma Yu-zhen [17] introduced a spindle roundness measurement method based on a capacitive scanning system that uses three capacitive displacement probes to eliminate spindle errors through averaging effects, thereby achieving roundness error separation and confirming the effectiveness of the measurement system. Bin Duan [18] proposed a novel roundness measurement method based on single-probe scanning that simplifies mathematical processing without requiring complex forward and backward Fourier transforms. Through simulation analysis, the method’s feasibility and repeatability were verified, and the roundness measurement’s accuracy was significantly improved. Jie Liang [19] introduced a method for measuring roundness using a laser profile scanner and merging the line point cloud data, using two-dimensional laser displacement sensors to collect line point cloud data and merging them radially and circumferentially to achieve micrometer-level high-precision measurement. Jiao Bai et al. [20] proposed a three-probe measurement method based on error separation technology. They used the Monte Carlo method to analyze the effects of sensor angle, off-axis distance, and harmonic suppression on error separation. Using a spectral confocal displacement sensor for roundness measurement, high-precision measurements in the sub-micron range were achieved. Erik Oertel [21] proposed a roundness measurement method based on an atomic force microscope, which enables the high-precision measurement of small, complex components through surface scanning and stitching algorithms. Eiki Okuyama [22] proposed a combined method, using the three-point method for roundness contour measurement and the integral method for straightness contour measurement. Through the three-point method, the roundness contour of the workpiece and the radial movement of the rotary table were separated, and reverse filtering was used to restore the original roundness contour, thus ensuring measurement stability. A Küng [23] proposed a roundness measurement method based on a three-dimensional coordinate measuring machine, reducing the motion mass and ensuring low isotropic stiffness through the innovative design of the flexible hinge–parallel motion structure, further improving the roundness measurement accuracy. These methods include the use of rotary scanning roundness testers, two-point or three-point methods, and coordinate measuring machines. Although these methods have proven effective in many cases, the influence of workpiece eccentricity on roundness measurement becomes more pronounced when measuring small cylindrical workpieces, especially when the diameter is less than 3 mm, making accurate measurement unattainable [24]. The non-contact measurement methods proposed by relevant scientists, particularly that of roundness measurement using spectral confocal technology, can eliminate the errors introduced by contact-based measurements, improving measurement accuracy [25,26,27,28]. However, accurate measurement remains a challenge, especially when dealing with small, weakly reflective objects. Therefore, research into contact-based methods remains crucial. To overcome the difficulties of measuring small-sized workpieces, relevant teams have proposed a stitching linear scan method using hardware filters [29,30,31,32,33,34]. The efficient filtering of high-frequency data and its impact mitigation have been achieved by replacing a diamond stylus with a tip radius of 2 μm with a ruby stylus with a tip radius of 150 μm. This substitution has allowed for the precise measurement of roundness in cylindrical rollers, which have dimensions of 10 mm in length and 1.5 mm in diameter. A roundness profile was reconstructed by splicing a series of circular arcs to derive roundness values, thereby facilitating the high-precision measurement of small cylindrical workpieces. This method has been shown to have exceptional measurement accuracy and repeatability for several critical measurement outcomes during the assessment of small-sized workpieces, in contrast to the conventional stitching linear scan method. Therefore, it is essential to investigate how using different-sized styluses impacts the results of workpiece roundness measurement through comparative analysis, especially for small-sized workpieces.

In this paper, experiments in roundness metrology were conducted on a small cylindrical workpiece, 1.5 mm in diameter and 5.8 mm in length, to investigate the efficacy of the stitching linear scan method for measuring roundness, with both diamond and ruby ball styluses employed, respectively. Quantitative analyses were conducted using the cross-correlation function, Euclidean distance, residual sum of squares, position error, and curvature. The measuring performance was evaluated by comparing the results obtained with the ruby ball stylus to the results obtained with the diamond stylus.

## 2. Principle and Experiments

**Figure 1:**
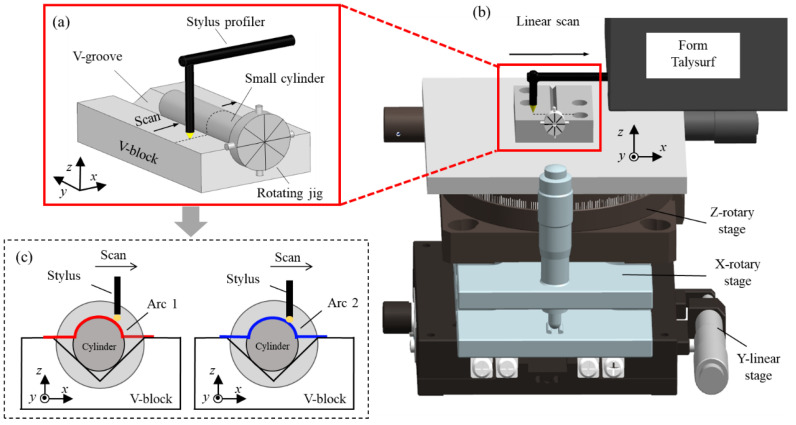
The linear scan on the surface of the needle roller by a profilometer: (**a**) The stylus scans across the surface of the needle roller; (**b**) the schematic of linear scan by a profilometer; (**c**) the arc coordinate data obtained by the stylus scanning.

**Figure 2:**
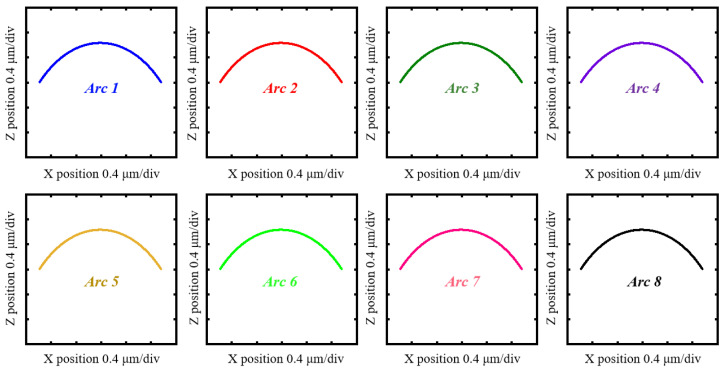
The eight arc coordinate data obtained by the linear scan of the profilometer.

The surface topography of the measured needle roller on a nano-scale can be conceptualized as a form which can be reconstructed with coordinates. Thus, a profilometer is employed to obtain these coordinates. As shown in Figure 1b, a Taylor Hobson Form Talysurf PGI 420 (VirtualExpo, Marseille, France) roughness measuring machine, with specifications shown in Table 1, is used to perform the experiments. As shown in Figure 1a, the needle roller is attached with a rotating jig and positioned on a V-block mounted on a multi-degree-of-freedom stage. Prior to conducting experiments, alignment through the adjustment of the stage’s position around the X and Z axes must be achieved. As shown in Figure 1c, the stylus of the roughness measuring machine scans across the needle roller surface and returns to the initial position. Arc coordinate data can be obtained. Subsequently, the rotating jig is manipulated to rotate 45°, causing the needle roller to also rotate by 45°, after which the stylus performs a second scan to obtain the subsequent arc coordinate data. This process is repeated until the entire cross-section circle has been scanned. As shown in Figure 2, eight arc coordinate data are obtainable. Of note, there is an overlapping portion for adjacent arc stitching.

**Figure 3:**
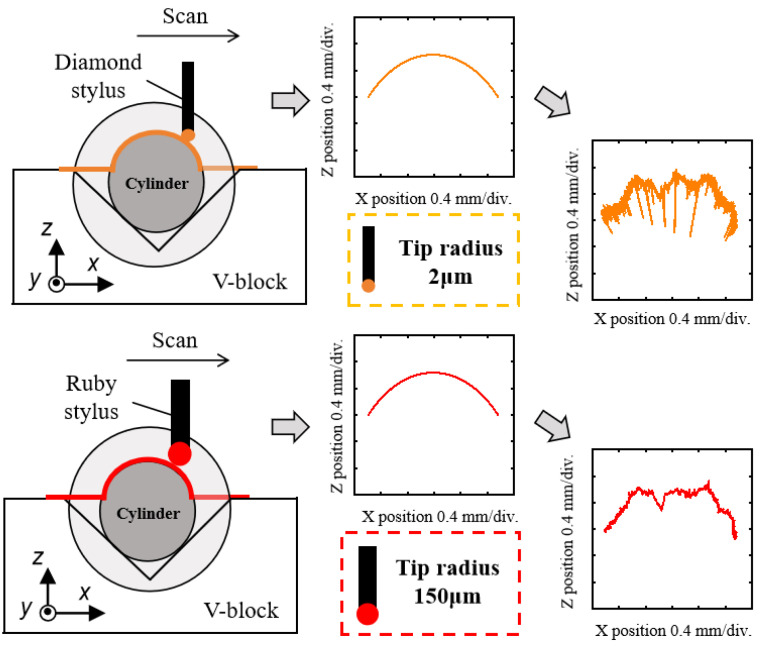
The scheme of linear scanning with different ball sizes.

**Figure 4:**
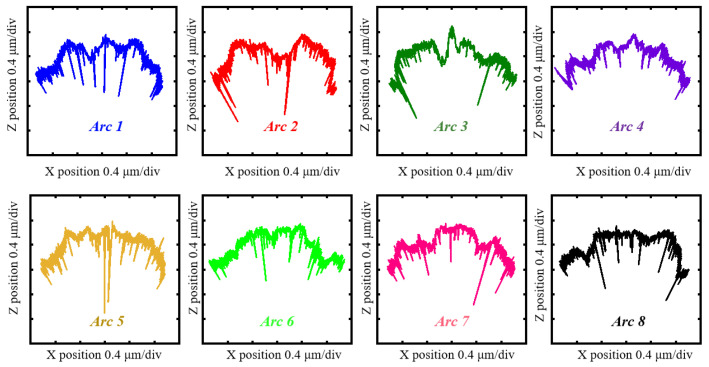
The eight arc profiles characterized according to the arc coordinate data obtained using the diamond stylus.

**Figure 5:**
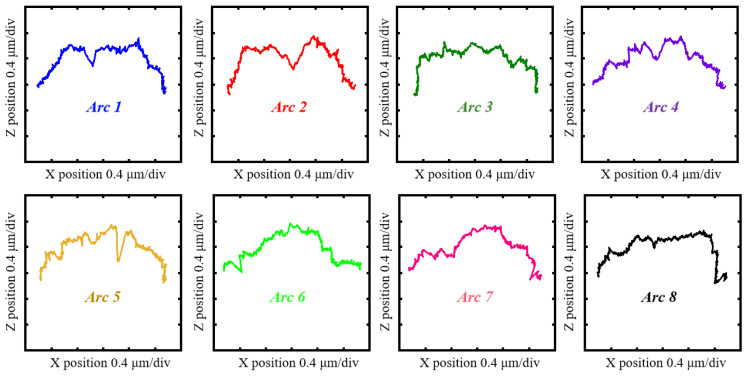
The eight arc profiles characterized according to the arc coordinate data obtained using the ruby ball stylus.

As shown in Figure 3, two sets of experiments were conducted under identical conditions. One employed a diamond stylus with a tip radius of 2 μm, while the other used a ruby ball stylus with a tip radius of 150 μm. This enabled the characterization of arc profiles through data processing. Figure 4 and Figure 5 show the eight arc profiles characterized according to the arc coordinate data obtained using a diamond stylus and a ruby ball stylus, respectively.

**Figure 6:**
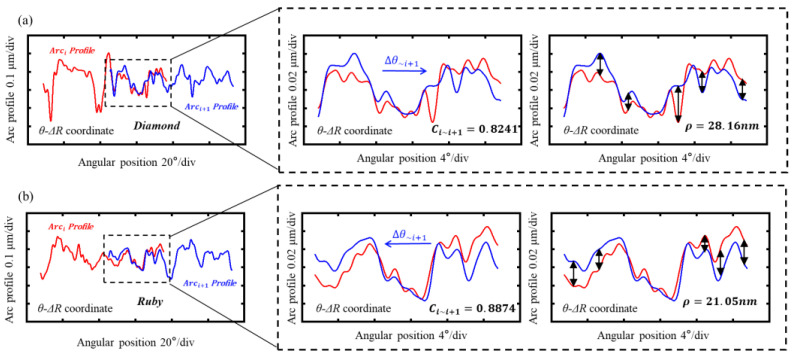
The matching of adjacent arc profiles: (**a**) the matching of adjacent arc profiles from diamond stylus; (**b**) the matching of adjacent arc profiles from ruby ball stylus.

The roundness profile of the needle roller is effectively reconstructed through the assembly of the eight arc profiles. Figure 6 shows the arc profile transformation, from the X-Z to the θ−ΔR coordinate system, which enhances the visualized matching of adjacent arc profiles. Figure 6a,b show the matching of adjacent arc profiles from the diamond and ruby styluses, respectively. The cross-correlation function serves to evaluate the circumferential matching of the overlapping portions between adjacent arc profiles, as defined in Equation (1). In this context, C represents the coefficient, g(θ) denotes the plotted arc profile, and θ denotes the angular position of the arc profiles. The Euclidean distance, as defined by Equation (2), evaluates the radial matching for the overlapping portion between adjacent arc profiles, where Δr is the difference between the radius and the reference radius in accordance with each coordinate on each arc coordinate data.

(1) $$C_{i\sim i+1}=\int_{-\infty }^{\infty }g_{i}\left(\theta \right)g_{i+1}\left(\theta +\Delta \theta_{i\sim i+1}\right)d_{\theta }$$

(2) $$D_{i\sim i+1}=\frac{1}{n_{c}}\sum_{i,j=1}^{n_{c}}\sqrt{{\left(\theta_{i,j}-\theta_{i+1,j}\right)}^{2}+{\left(\Delta r_{i,j}-\Delta r_{i+1,j}\right)}^{2}}$$

In general, a higher coefficient value is indicative of a superior circumferential alignment, while a smaller Euclidean distance is indicative of an improved radial alignment.

**Figure 7:**
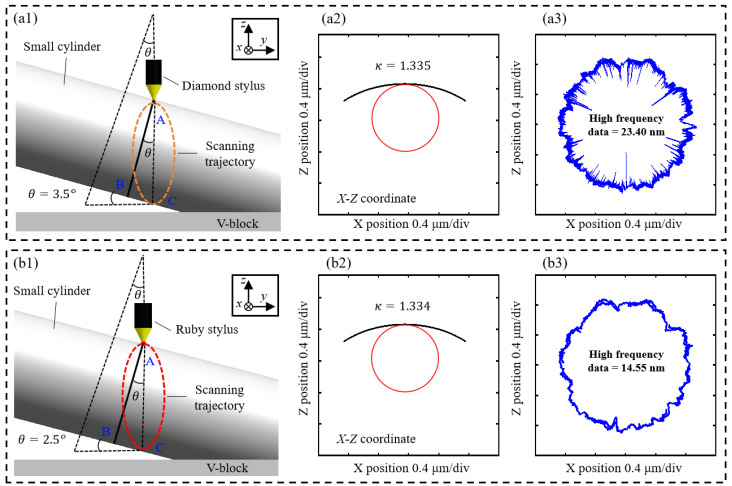
The evaluation of arc coordinate data quality: (**a1**,**b1**) the position error when scanning; (**a2**,**b2**) the curvature of the arc obtained; (**a3**,**b3**) the high-frequency data of the arc obtained.

Only if both the circumferential matching and radial matching are good may the matching be considered good. The roundness profile and its corresponding value are affected by two types of roundness matching. A position error, $\theta_{E}$, occurs during the scanning with the diamond and ruby ball styluses, as shown in Figure 7(a1,b1). Therefore, the trajectory of the stylus on the surface of the needle roller is not always a circle but an ellipse. The position error can be calculated by Equation (3), where $d_{mean}$ is the small cylinder’s diameter and $a_{ellipse}$ is the ellipse’s long axis.

(3) $$\theta_{E}=d_{mean}/a_{ellipse}$$

The quality of the eight arc coordinate data can be evaluated by calculating the mean curvature K, as shown in Figure 7(a2,b2). The high-frequency data that can affect the stitching of the adjacent arc profile, as well as the final roundness profile, can be calculated by the residual sum of squares between the reference radius and radius in accordance with each coordinate on each arc coordinate data, as shown in Figure 7(a3,b3).

## 3. Comparison Analysis

**Figure 8:**
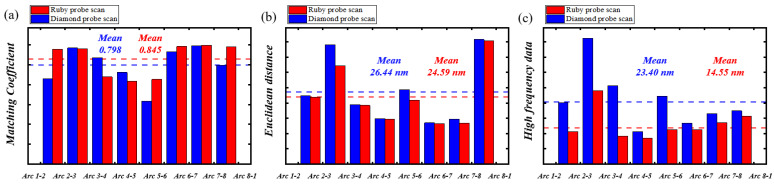
The evaluation results of the diamond stylus and ruby ball stylus: (**a**) the histogram of matching coefficient; (**b**) the Euclidean distance; (**c**) the high frequency data.

Two sets of roundness measurements were performed using a ruby ball and a diamond stylus, respectively, and the measurement performance of the two styluses was quantitatively analyzed using the evaluation methods described above. Figure 8a shows the coefficient for adjacent arc profiles, characterized by the arc coordinate data from the ruby ball stylus, which has a mean matching value of 0.845. This value is lower than the 0.798 coefficient obtained from the diamond stylus, indicating a superior circumferential alignment. Figure 8b shows that the mean Euclidean distance for the overlapping portions of adjacent arc profiles, characterized by the arc coordinate data scanned using the diamond stylus, is 26.44 nm, which is greater than the 24.59 nm with respect to the ruby ball stylus. This indicates that the stitched roundness profile obtained with the diamond stylus exhibits a greater degree of deviation compared to that obtained when using the ruby ball stylus. Figure 8c shows that the high-frequency data of the eight arc profiles with respect to the ruby ball stylus are 14.55 nm, which is less than the result of 23.40 nm with respect to the diamond stylus, indicating a smoother measured profile. As the fitting arc coordinates obtain the arc profiles, the curvature and position errors can reflect the data from the eight arc coordinates.

**Figure 9:**
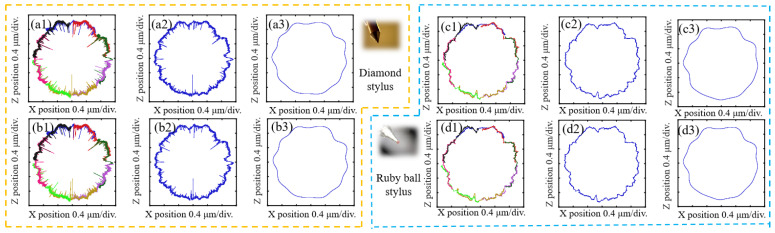
Results comparison: (**a1**–**a3**,**b1**–**b3**) results from two experiments using the diamond stylus; (**c1**–**c3**,**d1**–**d3**) results from two experiments using the ruby ball stylus.

The accuracy of the stitching of a roundness profile can be affected by a number of factors, including the matching coefficient of adjacent arcs, the Euclidean distance of overlapping portions of arc profiles, and the high-frequency data of the profiles, with these quantitative results presented in Table 2 for comparison. Figure 9 shows the final roundness profiles for the needle roller, with dimensions 1.5 mm in diameter and 5.8 mm in length. Figure 9(a1,b1) show the stitched roundness profile, as measured by the diamond stylus. Figure 9(a2,b2) show the roundness profile following the integration of the overlapping portion of adjacent arc profiles. Figure 9(a3,b3) show the roundness profile at the 50UPR filter. Similarly, Figure 9(c1–c3,d1–d3) show the roundness profiles with respect to the ruby ball stylus. Table 3 presents the quantitative data, which show that the ruby ball stylus’s measurement data outperforms that of the diamond ball stylus.

## 4. Conclusions

A roughness measuring machine was employed in experiments on the roundness metrology of a needle roller measuring 1.5 mm in diameter and 5.8 mm in length, using the stitching linear scan method. The needle roller’s roundness profile was reconstructed by stitching a series of arc profiles, according to the arc coordinate data obtained using a diamond stylus with a 2 μm tip radius and a ruby ball stylus with a 150 μm tip radius. The position error of the measurement and the curvature of the obtained arc were used to evaluate the arc coordinate data quality. The mean matching coefficient of adjacent arc profiles, the mean Euclidean distance of overlapping portions of adjacent arc profiles, and the high-frequency data were calculated to evaluate the roundness profile stitching quality. The results of the evaluation show that the arc coordinate data and roundness profile, with respect to the ruby ball stylus, demonstrate greater accuracy compared to the corresponding data from the diamond stylus. This indicates that the ruby ball stylus is more effective for measuring the roundness of small-sized needle rollers. The final roundness measurements of the needle roller, as determined by the diamond stylus and the ruby ball stylus, were found to be 0.16 μm and 0.14 μm, respectively. However, as the sole focus of this experiment was on the roundness measurements of a small needle roller with a diameter less than 3 mm and a length less than 50 mm, using styluses with different tip radii, other parameters, such as the stylus material, scanning speed, workpiece radius, and scanning rate, were not examined. Consequently, the inferences derived from this study are subject to certain constraints. In the future, experiments will be conducted to explore the performance of different styluses in measuring the roundness of rollers of various sizes. Additionally, experiments will be conducted to integrate other parameters, including conducting experiments on small-sized workpieces using large-sized styluses at low sampling rates.

## Figures and Tables

**Table 1:** The specification of Form Talysurf PGI 420.

Item	Resolution (Z)	Measuring Range (Z)	Measuring Range (X)	Sampling Step (X)	Scanning Speed
Value	3.2	4	120	0.125	0.1
Unit	nm	mm	mm	μm	mm/s

**Table 2:** The evaluation results.

	Matching Coefficient	Euclidean Distance	High-Frequency Data	Position Error	Mean Curvature
Diamond stylus	0.78	26.44 nm	23.40 nm	3.5°	1.335
Ruby ball stylus	0.85	24.48 nm	14.55 nm	2.3°	1.334

**Table 3:** The final diameter and roundness results.

	Tip Radius	Diameter of Needle Roller	Roundness of Needle Roller
Diamond stylus	2 μm	1.497 mm	0.16 μm
Ruby ball stylus	150 μm	1.499 mm	0.14 μm

## Data Availability

Data are contained within the article.
